# Unraveling the binding characteristics of small ligands to telomeric DNA by pressure modulation

**DOI:** 10.1038/s41598-021-89215-2

**Published:** 2021-05-06

**Authors:** Rosario Oliva, Sanjib Mukherjee, Roland Winter

**Affiliations:** Physical Chemistry I-Biophysical Chemistry, Faculty of Chemistry and Chemical Biology, TU Dortmund University, Otto-Hahn Strasse 4a, 44227 Dortmund, Germany

**Keywords:** Biophysics, Chemistry

## Abstract

Recently, non-canonical DNA structures, such as G-quadruplexes (GQs), were found to be highly pressure sensitive, suggesting that pressure modulation studies can provide additional mechanistic details of such biomolecular systems. Using FRET and CD spectroscopy as well as binding equilibrium measurements, we investigated the effect of pressure on the binding reaction of the ligand ThT to the quadruplex 22AG in solutions containing different ionic species and a crowding agent mimicking the intracellular milieu. Pressure modulation helped us to identify the different conformational substates adopted by the quadruplex at the different solution conditions and to determine the volumetric changes during complex formation and the conformational transitions involved. The magnitudes of the binding volumes are a hallmark of packing defects and hydrational changes upon ligand binding. The conformational substates of the GQ as well as the binding strength and the stoichiometry of complex formation depend strongly on the solution conditions as well as on pressure. High hydrostatic pressure can also impact GQs inside living cells and thus affect expression of genetic information in deep sea organisms. We show that sub-kbar pressures do not only affect the conformational dynamics and structures of GQs, but also their ligand binding reactions.

## Introduction

In recent years, G-quadruplexes (GQs) have attracted considerable attention among the various noncanonical DNA structures because of their unique conformations, gene functions as well as potential targets for chemical intervention of biological function^1–4^. G-quadruplexes are four-stranded nucleic acid structures formed by guanine-rich sequences where G-tetrads are formed by coplanar arrangement of four Hoogsteen-paired guanines and are known to be highly polymorphic in nature^5–8^. GQs present in living cells are mostly found in mitochondrial DNA, RNA, and key regions of the human genome, such as gene promoters (e.g., c-myc, bcl-2) and telomeres regulating several cellular functions, like gene transcription and telomere lengthening^6–12^. Further, they are involved in important cancer-related biological processes and hence acquired particular attention among non-canonical nucleic acid motifs^1–5,10,11^. The human telomeric G-quadruplex is known to take up several structures, like antiparallel, parallel, and hybrid conformations, depending on the monovalent cationic salt (Na^+^ and K^+^), osmolyte concentration, as well as on the physical parameters temperature and pressure^6,13–15^. Due to their significant abundance in functional genomic regions and in the untranslated regions of mRNAs, GQs have been recognized as a potential drug target to stop telomeric function and regulate gene expression^5,10,16,17^. Zahler et al. found that telomeric activity is significantly reduced upon folding of telomeric DNA into a G-quadruplex^18^. Such properties of GQs raised keen interest toward small molecules that are able to bind to GQ structures and alter the telomere maintaining mechanism^3,19^. Several ligands that bind to and modulate the function of GQs have been identified as chemotherapeutic agents^20–23^. A particular focus has been on small ligands that stabilize GQ structures, thereby altering telomeric function in cancer cells which results in inhibition of tumor growth^24,25^. As prerequisite for such biomedical applications, a comprehensive understanding of the binding of the small molecule ligands to GQs is required.

Among different quadruplexes, the human telomeric motif d[AG_3_(T_2_AG_3_)_3_], which consist of tandem repeats of the TTAGGG sequence followed by a terminal 3′ G-rich single-stranded overhang of 150–200 nucleotides in length, has immense importance because of its direct relevance for inhibiting telomerase activity^10,16,17^. The 22AG, an oligonucleotide that mimics the human telomeric motif has been well characterized^26^ and is used for this study. The 22AG contains four repeats of the human telomeric motif (TTAGGG) and has been observed to fold into different structural topologies, depending on the salt and other small molecules in solution. Earlier studies found that Na^+^ induces an antiparallel conformation, while the presence of K^+^ induces a hybrid-1 conformation^5,27^. It has also been found that many of the quadruplex-binding dyes interact with DNA in a non-specific manner^5,28,29^. In the present study we have used a well-known fluorescence dye as ligand, thioflavin T (ThT), which is also excessively used for the detection of amyloid fibrils in protein aggregation pathways. As the fluorescence intensity of ThT is significantly enhanced after binding to the 22 AG human telomeric DNA^5,30^, the conformational dynamics and ligand binding properties of this quadruplex motif can be readily studied, both in dilute solution as we as in cell-mimicking crowded environments.

Although high-pressure studies have become a common biophysical technique and have been shown to reveal important additional information about thermodynamic and kinetic parameters of biomolecular systems^31–38^, pressure studies on ligand binding of nucleic acids are still *terra incognita* and hence deserve particular attention. A significant portion of the global biosphere exists at a depth of more than 1000 m and is therefore subject to high hydrostatic pressure (HHP) conditions of several hundreds of bars^39,40^. Hence, the biological relevance of high-pressure biophysical studies lies in understanding the physiology and adaptation mechanism of deep-sea organisms living under such harsh environmental conditions. Apart from this biological relevance, high-pressure studies on biomolecular systems are also important from the physico-chemical point of view^31–34^. Pressure-axis experiments can be carried out to provide additional details of the free-energy and conformational landscape of biomolecules, and were in particular successful in uncovering conformational substates of the biomolecular systems^31–37^. According to Le Châtelier's principle, an increase of pressure shifts the conformational equilibrium of the system towards states with smaller partial molar volume. The pressure effect on a given reversible reaction follows the relation (dln*K*/d*p*)_*T*_ = −Δ*V*/(*RT*), where *K* is the equilibrium constant and ∆*V* is the associated volume change of the reaction (e.g., a conformational transition), which sensitively depends on the packing properties (incl. void volume) and hydration properties of the system^31–38^. Regarding nucleic acid systems, application of pressure has been shown to slightly decrease the Watson–Crick H-bond distance, favor base stacking interactions, and to generally stabilize the double stranded B-DNA structure^31,37,41^. However, it has recently been found that noncanonical DNA structures, such as G-quadruplex DNA, are highly sensitive to pressure^15,36,38^, suggesting that pressure modulation studies can provide additional mechanistic details of such biomolecular systems. We applied the pressure-perturbation approach in this study to reveal novel information about the conformational fluctuations and structural transformations as well as thermodynamic properties (volume and hydration changes) of the 22AG quadruplex upon ligand binding.

Macromolecular crowding as encountered in the biological cell (in the total concentration range 300–400 mg mL^−1^) is expected to modulate the structural properties of the GQ conformers and the binding characteristics of the ligand-GQ system. Hence, experiments have been carried out not only in different salt environments but also in the presence of the macromolecular crowding Ficoll 70 in the attempt to clarity how GQs and their binding properties are affected under cell-mimicking conditions. Generally, the effect of inert macromolecular crowders results in a reduction of exposed solvent accessible surface area and a hence a shift of conformational equilibria toward the more compact state^42^. As shown recently, confinement by macromolecular crowding can lead to remarkable changes in the conformation of GQs^36,43–45^.

## Materials and methods

### Materials

The G-quadruplex forming DNA sequence used in this study (22AG) and the labelled one were purchased from GenScript (Leiden, Netherlands). The sequence is: [dA(GGGTTA)_3_GGG]. The labelled sequence has the fluorophores carboxyfluorescein (FAM) in 5′ and 5-(and-6)-carboxytetramethylrhodamine (TAMRA) in position 3′ attached. The salts sodium chloride (NaCl) and potassium chloride (KCl) were purchased from Sigma Aldrich Chemicals. The crowding agent Ficoll 70, thioflavin T (ThT), and the Tris–HCl for buffer preparation were also purchased from Sigma Aldrich Chemicals. All the reagents were used without further purification.

### Sample preparation

The pressure stable Tris–HCl buffer was prepared at a concentration of 30 mM at the final pH of 7.4 by using deionized water. The buffers containing 60 mM NaCl or KCl were prepared by dissolving an appropriate amount of the salts in buffer. The buffer containing the crowding agent Ficoll 70 was prepared by dissolving it in Tris–HCl buffer. The final concentration of Ficoll in the samples was 25 wt%. A concentrated stock solution of 22AG was prepared by dissolving it in Tris–HCl buffer. The concentration was determined by measuring the absorbance of the solution at 260 nm, using the extinction coefficient^5^ of 228,500 M^−1^ cm^−1^. The absorbance was evaluated at 90 °C where the DNA sequence is completely unfolded by using a full volume 1-cm path length quartz cuvette. The stock solution of ThT was instead prepared in water. Its concentration was evaluated at 412 nm where the extinction coefficient^30^ is 36,000 M^−1^ cm^−1^.

### Fluorescence spectroscopy—ligand binding

The interaction of ThT with 22AG was followed using steady-state fluorescence spectroscopy. The experiments were performed by means of a K2 fluorimeter from ISS (Champaign, IL, USA). A solution of ThT at the concentration of 2.14 µM was titrated with 22AG in the range 0 µM to 45 µM. The experiments were carried out at 25 °C in 30 mM Tris–HCl buffer and in the same buffer with the addition of 60 mM NaCl or 60 mM KCl or 25 wt% Ficoll. The wavelength of excitation was 425 nm. The fluorescence emission was collected in the range 450–550 nm. The slits for the excitation and emission monochromators were both set to 16 nm. For the high hydrostatic pressure (HHP) experiments, a high-pressure cell system from ISS and quartz cuvettes were used. The pressure was controlled by means of a manual pump and water was used as pressurizing fluid. A pressure range from 1 to 2000 bar was covered. The samples were loaded in the cuvette, sealed with DuraSeal™ laboratory stretch film, and placed into the high-pressure cell. In order to determine the binding constant (*K*_b_), a plot of Δ*F vs.* total 22AG concentration was made. Here, Δ*F* = *F* *−* *F*_0_, where *F* and *F*_0_ are the fluorescence intensities of ThT in the presence and in the absence of 22AG, respectively. The data were fitted with an *n* equivalent and independent binding sites model, as described in detail in^46^.

### Fluorescence spectroscopy—Job’s plot

The method of continuous variation (or Job’s plot) is a model-free method which allows to determine binding stoichiometries^47–50^. Briefly, solutions containing ThT and 22AG at the total concentration of 35 µM were prepared. Then, the ThT mole fraction (*x*_ThT_) was varied between 0 and 1. For each sample, the fluorescence intensity was recorded in the range 450–550 nm and in the pressure range 1–2000 bar, using the same experimental equipment as described above. The experiments were performed in 30 mM Tris–HCl, pH 7.4, and in the same buffer containing 60 mM NaCl or 25 wt% Ficoll at the temperature *T* = 25 °C.

### Fluorescence spectroscopy—FRET

Fluorescence resonance energy transfer (FRET) experiments were performed in the pressure range 1–2000 bar and with the same equipment as described above. For these experiments, labelled 22AG was used. Briefly, solutions of labelled 22AG at the concentration of 2 µM in the absence and in the presence of 20 µM ThT were prepared. The excitation wavelength was set to 485 nm. The fluorescence emission was recorded in the range 500–650 nm. The experiments were carried out in 30 mM Tris–HCl, pH 7.4, and in the same buffer containing 60 mM NaCl or 60 mM KCl or 25 wt% Ficoll at *T* = 25 °C. The relative FRET efficiency (*E*_rel_) was calculated by using *E*_rel_ = *I*_A_/(*I*_A_ + *I*_D_)^35^, where *I*_D_ and *I*_A_ are the fluorescence intensities at the maximum of the donor (FAM) and the acceptor (TAMRA), respectively.

### Circular dichroism

The circular dichroism (CD) of quadruplexes arises primarily from the stacking arrangement of the guanine bases within G-quadruplex stacks. Hence, CD spectroscopy can be employed to determine the conformations adopted by 22AG in the different media and in the presence of ThT. CD experiments were carried out by means of a J-715 spectropolarimeter from Jasco Corporation (Tokyo, Japan) at the temperature of 25 °C, using a quartz cuvette with a path length of 0.1 cm in the range 220–320 nm. Solutions of 30 µM 22AG in the absence and in the presence of 300 µM ThT were prepared. The experiments were carried out in 30 mM Tris–HCl, pH 7.4, and in the same buffer containing 60 mM NaCl or 60 mM KCl or 25 wt% Ficoll. The following instrumental parameters were used: scan rate of 50 nm min^−1^, response time of 2 s, and band width of 5 nm. Each spectrum is the results of at least 3 accumulations. For each sample, a background blank (working buffer with and without ThT) was subtracted. The spectra were normalized per molar concentration of single strand and path length (Δ*ε/*M^−1^ cm^−1^).

## Results

### Binding of ThT to 22AG at ambient conditions

**Figure 1:**
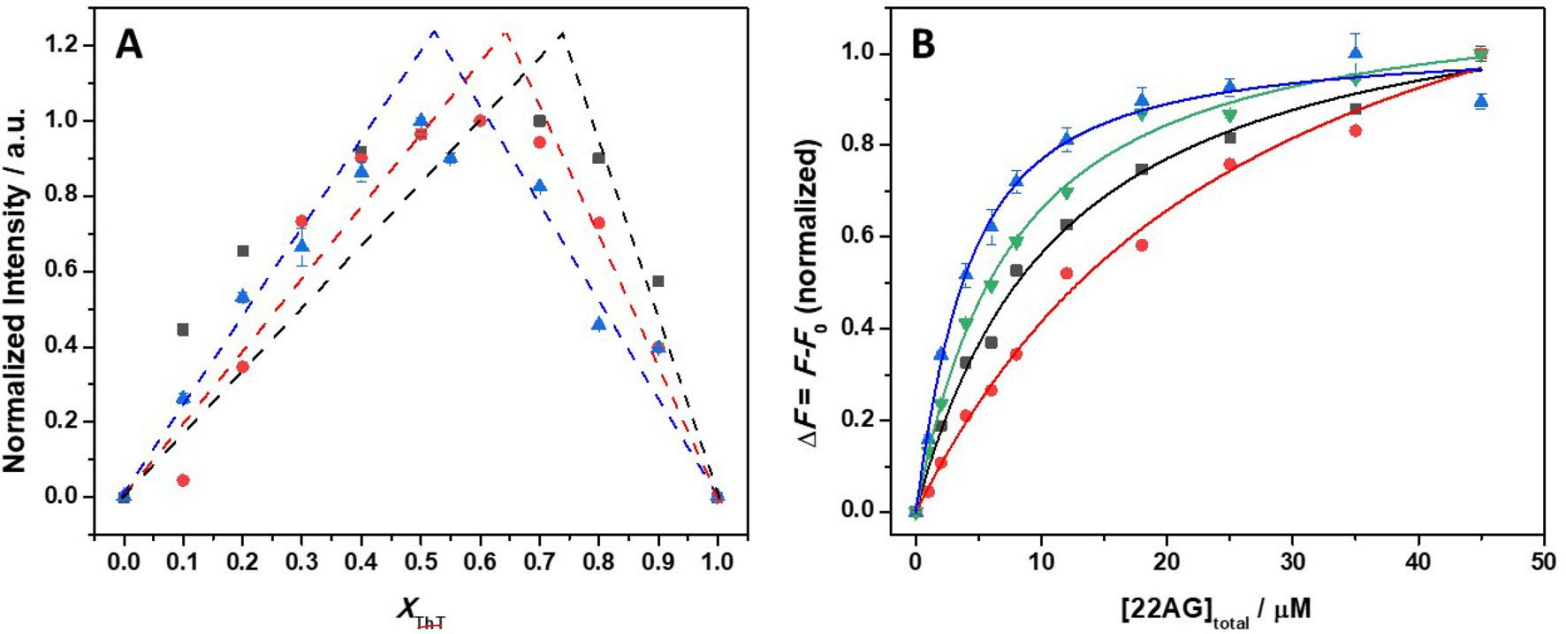
(**A**) Job’s plot for the ThT/22AG system in 30 mM Tris, pH 7.4 (black squares) and in the same buffer with 25 wt% Ficoll (red circles) and in the presence of 60 mM NaCl (blue triangles). For comparison, the dashed lines represent the positions of 1:1, 1:2 and 1:3 (22AG:ThT) complexes in an ideal case where only one type of complex is present in solution. (**B**) Binding isotherms for the titration of a 2.14 µM ThT solution with a solution of 22AG in 30 mM Tris, pH 7.4 (black squares), in the same buffer with the addition of 60 mM NaCl (red circles), in the presence of 60 mM KCl (blue triangles), and in the presence of 25 wt% Ficoll (green reversed triangles). To be able to compare these data, the binding isotherms were normalized. All the experiments were carried out in triplicate at ambient conditions (*p* = 1 bar and *T* = 25 °C). Where not shown, the error bars are smaller than the symbol size.

In previous works^5,30^, it was demonstrated that ThT can selectively bind to G-quadruplexes, such as the human telomeric 22AG sequence used in this study, with an affinity that strongly depends on the solution conditions, such as the presence of monovalent cations. ThT exhibits a very low fluorescence signal when dissolved in buffer solution^5^. Instead, upon interaction with 22AG, a strong fluorescence intensity is observed. A similar behavior was also observed for thiazole orange binding to 22AG quadruplex^51^. Thus, fluorescence spectroscopy can be used to follow the formation of the ThT/22AG complex and for the determination of the binding constant (*K*_b_) in the different media and as function of high hydrostatic pressure (HHP). In order to determine the value of *K*_b_, the experimental data need to be fitted with an appropriate binding model with a given stoichiometry. In previous work, it was demonstrated that in the presence of Na^+^ and K^+^, one ThT is bound to one 22AG at ambient conditions^30^. We applied the method of continuous variation (also denoted as Job’s plot) to determine the number of ThT bound to 22AG also in pure buffer (i.e., in the absence of any cations) and in the presence of 25 wt% Ficoll. Figure 1A shows the Job’s plot at ambient condition (*p* = 1 bar, *T* = 25 °C) for the complex formation in 30 mM Tris, pH 7.4, and in the same buffer in the presence of 25 wt% Ficoll. For comparison, the Job’s plot in the presence of 60 mM NaCl is also reported.

The Job’s plot is a model-free method that allows the determination of the stoichiometry of a given complex^47^. A series of solutions are prepared such that the total concentration (i.e. [ThT] + [22AG]) of the interacting partners is constant, and at the same time the mole fraction (*x*_ThT_) is varied (for details, see the Materials and Methods section). To determine the binding characteristics, we recorded the fluorescence emission of ThT. Since ThT in the absence of 22AG is basically not fluorescent, the fluorescence signal is directly related to the complex formation. Abrupt changes in the Intensity *vs. x*_ThT_ plots indicate the stoichiometry of the complex formed. Though this method is attractive for its simplicity, the accuracy and the identification of multiple complexes present in solution have to be taken with care^49,50^. For ideal cases, where only one type of complex is present (dashed lines in Fig. 1A), only one inflection point is present. The Job’s plots reported in Fig. 1A clearly show that in pure buffer and in the presence of the crowder Ficoll, the binding stoichiometry is not 1:1. For comparison, the experiment performed in the presence of NaCl is also reported where the formation of a 1:1 complex is inferred from the inflection point at *x*_ThT_ = 0.5. An inspection of the data reveals that for pure buffer conditions, an inflection point around 0.7 is present, which is indicative of 2 or 3 ThT molecules bound to one 22AG. Instead, in the presence of Ficoll, the inflection point appears around 0.6, suggesting that 2 ThT molecules are bound to one molecule of 22AG. Thus, a comparison among the reported Job’s plots reveals that the most likely stoichiometries are (22AG:ThT): 1:3, 1:2 and 1:1 in pure buffer, in the presence of Ficoll, and in the presence of Na^+^, respectively. The formation of a 1:1 complex in the presence of K^+^ salt was already demonstrated^30^.

Figure 1B depicts the binding isotherms for the complex formation in all four media at ambient conditions. The data are well fitted by means of an equivalent and independent binding sites model^46^, i.e., it was assumed that when more than one ThT molecules are bound, they have the same affinity for the 22AG. The values of the binding constant and the stoichiometries determined from the fit are reported in Table 1.

**Table 1:** Binding constants and stoichiometries for the 22AG:ThT complex formation at various solution conditions (*p* = 1 bar, *T* = 25 °C).

Solvent	*K*_b_/M^−1^ ∙10^5^	22AG:ThT stoichiometry
30 mM Tris, pH 7.4	0.87 ± 0.06	1:3
+ 60 mM NaCl	0.42 ± 0.04	1:1
+ 60 mM KCl	3.51 ± 0.42	1:1
+ 25 wt% Ficoll	1.39 ± 0.07	1:2

An inspection of Table 1 reveals that the binding of ThT to 22AG depends strongly on the solution conditions and, thus, on the conformation(s) the nucleic acid strand adopts at these conditions. The ligand ThT has the highest affinity for 22AG in the presence of K^+^, followed by Ficoll, pure buffer, and finally the presence of Na^+^. The values of *K*_b_ in the presence of NaCl and KCl are in good agreement with previously reported data^30^, where similar binding constants were determined from fluorescence titration experiments. Using the stoichiometries derived from the Job 's plots, very good data fits could be obtained.

### The conformation of the 22AG/ThT complex at ambient conditions

**Figure 2:**
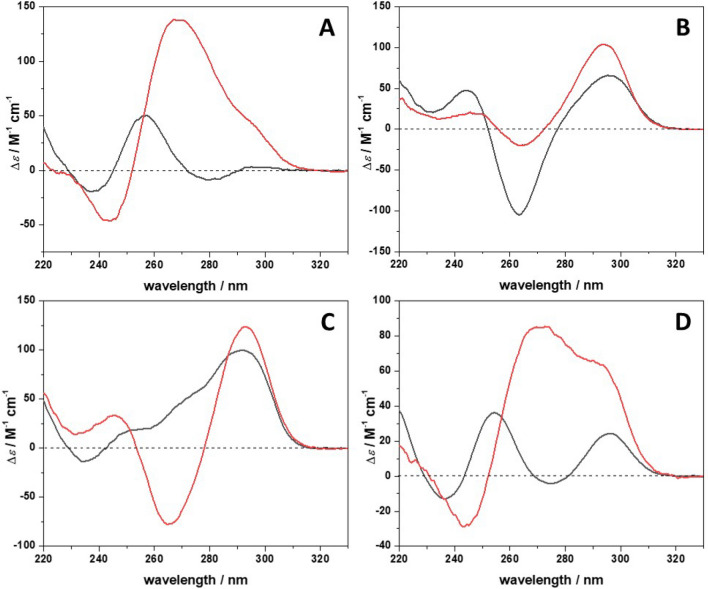
Circular dichroism (CD) spectra of a 30 µM solution of 22AG in the absence (black spectra) and in the presence of 300 µM ThT (red spectra) in (**A**) 30 mM Tris, pH 7.4, (**B**) in the presence of 60 mM NaCl, (**C**) in the presence of 60 mM KCl, and (**D**) in the presence of 25 wt% Ficoll. All experiments were carried out in a 0.1 cm path length quartz cuvette at the temperature of 25 °C.

To explore the effect of ThT binding on the conformation of 22AG, circular dichroism (CD) spectroscopy experiments were carried out. Figure 2 shows the CD spectra of 22AG in the four media and in the absence and presence of ThT at a ThT/22AG molar ratio of 10. In pure buffer, the sequence 22AG is mainly unfolded, as evidenced by the positive CD band at ~ 256 nm^52^. However, the weak positive band at ~ 295 nm indicates an additional small fraction of folded conformation. In the presence of ThT, a strong conformational change is detected. The spectrum is now characterized by a maximum at 269 nm with a shoulder at ~ 295 nm and a minimum at 243 nm, signifying a mixture of different conformations. The CD bands at 269 nm and at 243 nm are indicative of a parallel structure, whereas the presence of a band at 295 nm is probably due to the presence of a hybrid/antiparallel conformation^53,54^. Since the intensity of the band at 295 nm is much smaller with respect to that at 269 nm, the fraction of 22AG folded in the hybrid/antiparallel conformation is comparatively smaller with respect to the parallel one.

In the presence of 60 mM NaCl, the CD spectrum of 22AG exhibits a positive band at 295 nm and a negative one around 263 nm, which points to an antiparallel structure^5^. In the presence of ThT in the Na^+^-containing medium, the position of the positive and negative bands is the same, suggesting that no conformational changes occurred upon binding of the ligand. However, the intensities of the two bands, in particular of the negative ones, are not the same as in the absence of ThT. This suggests that even if the overall structure in both cases is antiparallel, minor differences between the two structures could be present (e.g., in the loop region, quartet stacking geometry, or strand orientation). A hybrid-1 structure is instead adopted by 22AG in the presence of K^+^^52^. This is evidenced by the positive band centered at 292 nm, a small positive band around 268 nm and a negative one at 235 nm. Upon addition of ThT, a distinct change in the conformation of the nucleic acid is observed. The position of the bands (positive at 293 nm and negative at 265 nm) suggests the formation of an antiparallel structure, as in the case observed in the presence of Na^+^. Panel D of Fig. 2 depicts the CD spectrum of 22AG in the Ficoll-containing buffer solution. The spectrum is characterized by two positive bands, at 254 nm and 296 nm. The first one is a feature of an unfolded structure, and the band at 296 nm could be a signature of the presence of hybrid/antiparallel conformations. Upon addition of ThT, a CD spectrum similar to the one obtained for the case in pure buffer was obtained, i.e. a mixture of parallel and hybrid/antiparallel conformations. However, in the present case, the intensity of the band at 294 nm is comparable to the intensity of the band at 270 nm, indicating that, most likely, the fraction of 22AG adopting a hybrid/antiparallel conformation is higher in the presence of Ficoll compared to that observed in pure buffer. Thus, multiple conformational states of the 22AG seem to be populated in the presence of the crowding agent.

### Effect of high pressure on the conformation of 22AG and the 22AG/ThT complex

**Figure 3:**
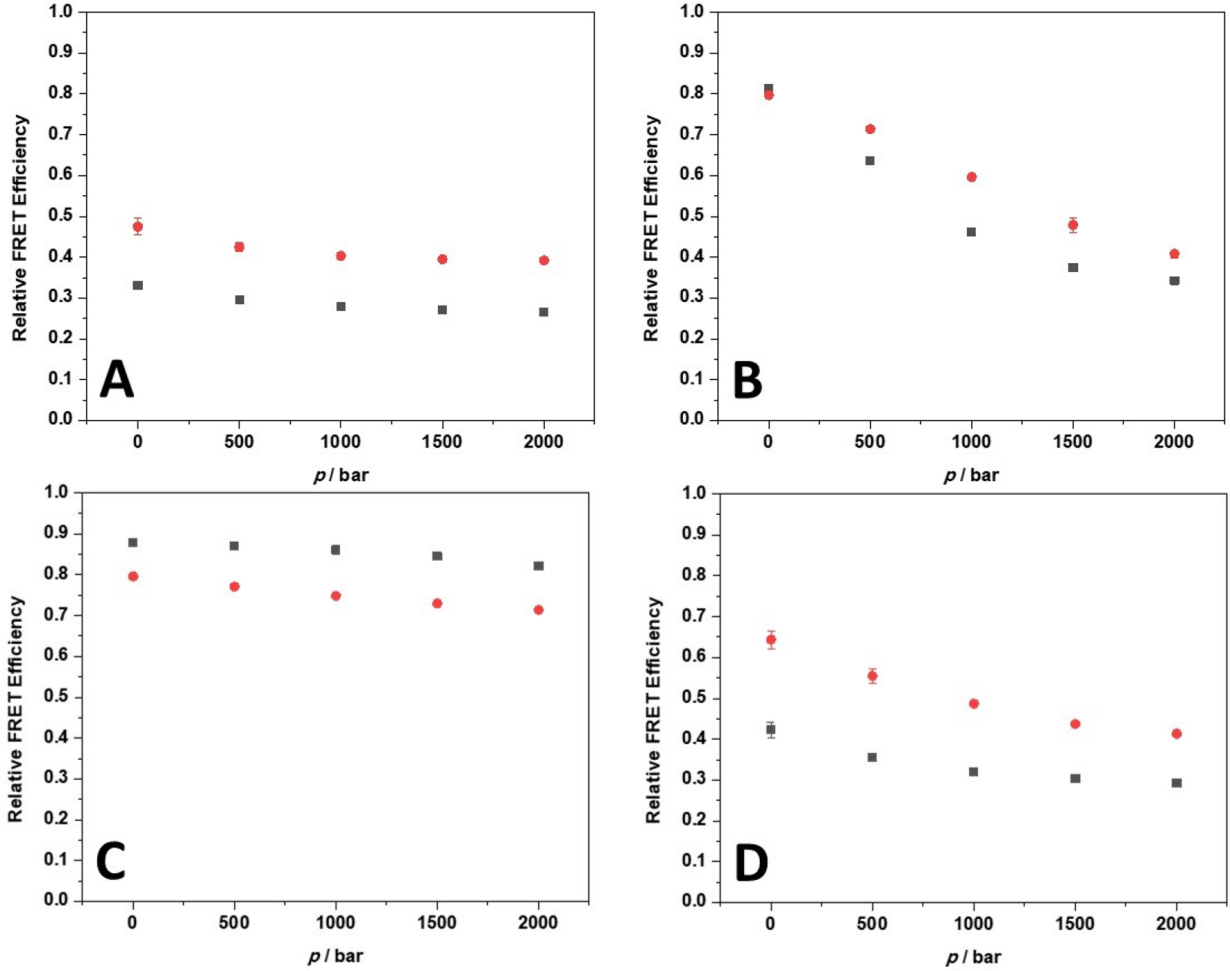
Plots of the relative FRET efficiency (*E*_rel_) as a function of pressure for the dually labelled 22AG in the absence (black squares) and in the presence (red circles) of ThT (ThT:22AG ratio = 10:1) in (**A**) 30 mM Tris, pH 7.4, (**B**) in the presence of 60 mM NaCl, (**C**) in the presence of 60 mM KCl, and (**D**) in the presence of 25 wt% Ficoll. All the experiments were carried out in triplicate at 25 °C. Where not shown, the error bars are smaller than the symbol size.

Before exploring the effect of HHP on the binding equilibrium between ThT and 22AG, it is mandatory to explore if pressure is able to change the conformation of the 22AG both in the absence and presence of ThT. To this end, conformation-sensitive high-pressure FRET spectroscopy experiments were performed using a dually labeled 22AG. The sequence was labeled with the fluorophores carboxyfluorescein (FAM) in the 5′ and with 5-(and-6)-carboxytetramethylrhodamine (TAMRA) in the 3′ position, which act as a FRET pair (FAM as donor, TAMRA as acceptor). The efficiency of energy transfer depends on the relative distance between the two fluorophores and thus, is sensitive to the conformation of the GQ adopted. When the two fluorophores are close to each other, higher values of the efficiency will be observed. Instead, for open (e.g., unfolded) conformations, the two fluorophores will be far from each other and, consequentially, the FRET efficiency will be lower. Figure 3 shows the FRET efficiency (*E*_rel_) of the labelled 22AG as a function of pressure for the four different solution conditions in the absence and presence of ThT. It is important to note that in our experiments there is no contribution from ThT fluorescence, since ThT is not excited at the employed wavelength (485 nm). The increase of the fluorescence intensity upon pressurization is due to the increase of the binding constant with increasing pressure, implying that the fraction of bound, fluorescent ThT is increasing with pressure. Intrinsic effects of pressure on the energy levels of the fluorophore and changes of the exited state deactivation processes leading to a change in quantum yield are rather small at these still rather low pressures.

The comparison of the FRET data obtained at 1 bar with the CD data reported in Fig. 2 allowed us to assign a certain *E*_rel_ value to a particular conformation. In addition, results from X-ray diffraction, NMR, CD, single-molecule FRET, and molecular dynamics simulation data of previous studies were used to assign the *E*_rel_-values to a particular conformation of the 22AG^5,13,15,27,55,56^. In pure buffer, an *E*_rel_ value of ~ 0.3 was observed. The corresponding CD measurements clearly indicate that 22AG is mainly unfolded. Thus, a value of *E*_rel_ ≤ 0.33 is indicative of an unfolded DNA structure. Instead, in the presence of NaCl, *E*_rel_ ≈ 0.81, which corresponds to a folded antiparallel structure of the 22AG. In the presence of KCl, the value of *E*_rel_ amounts to ~ 0.88 and the 22AG adopts a hybrid-1 conformation. For the assignment of *E*_rel_ to the parallel conformation, a comparison of the data reported in Fig. 3A for 22AG in the presence of ThT and the corresponding CD data in Fig. 2A can be used, which leads to an *E*_rel_ value around 0.47 for characterization of the parallel structure. Table 2 summarizes the tentative assignment of all 22AG conformations to their corresponding *E*_rel_ value.

**Table 2:** Relative FRET efficiency (*E*_rel_) for the various conformations of 22AG as inferred from the comparison of the FRET and CD data at *p* = 1 bar and *T* = 25 °C.


Conformation	*E*_rel_
Unfolded	~ 0.33
Antiparallel	~ 0.81
Parallel	~ 0.47
Hybrid-1	~ 0.88

The scheme at the top depicts the quadruplex structures adopted by the 22AG sequence: (A) the antiparallel, (B) the parallel, and (C) the hybrid-1 conformation. In the labeled sequences, the donor (FAM) is located at position 5′, and the acceptor (TAMRA) in position 3ʹ.

In pure buffer without any salts (Fig. 3A), the *E*_rel_ value at ambient conditions and in the absence of ThT is low, as expected for an unfolded structure. The application of pressure up to 2000 bar leads still to a decrease of *E*_rel_ from ~ 0.33 to ~ 0.26. It is known that HHP can cause unfolding of quadruplex structures. Thus, this further decrease of the FRET efficiency with pressure may be attributed to the presence of a small fraction of 22AG that even in pure buffer is not yet completely unfolded. Upon ThT binding to 22AG, the *E*_rel_ value increases slightly (from ~ 0.33 to ~ 0.47 at 1 bar), indicating, consistent with the CD measurements, a conformational change towards a parallel GQ conformation upon complex formation. The application of pressure to the complex causes small changes in *E*_rel_ only, indicating a high pressure stability and hence efficient packing of the complex (red squares in Fig. 3A).

In the presence of Na^+^ cations (Fig. 3B), the 22AG and 22AG/ThT complex at 1 bar exhibits a very similar and high value of *E*_rel_, demonstrating that upon ThT binding no conformational changes of the antiparallel structure occurs, in accordance with the CD data. In the absence of ThT, the application of the pressure causes a strong decrease of *E*_rel_, demonstrating that the antiparallel structure in Na^+^ containing medium is not pressure stable and a conformational change takes place. Since the value of *E*_rel_ at 2000 bar is similar to that observed in pure buffer at ambient conditions, we may conclude that the 22AG sequence is largely populating an unfolded state. As from ensemble FRET data it is not possible to unequivocally identify the population of the different conformeres, the relative FRET efficiency recorded here yields an average over all conformations of the molecule only. Instead, in the presence of ThT, the decrease of *E*_rel_ upon pressurization is less pronounced. A value of *E*_rel_ ≈ 0.41 is observed, suggesting that the 22AG is now mainly adopting a parallel conformation at high pressure conditions. A parallel conformation has also been observed for the ThT-GQ complex in pure buffer solution in the pressurized state. Mixed conformations of antiparallel folded and unfolded states cannot be excluded, however.

In the presence of 60 mM K^+^, the *E*_rel_ value of 22AG at 1 bar and in the absence of ThT is very high (~ 0.88), which points to a well folded structure, again in agreement with the CD measurements, which designates a hybid-1 structure. In the presence of ThT, *E*_rel_ drops to ~ 0.80, a value very similar to that observed in NaCl, indicative of an antiparallel structure of the 22AG/ThT complex. As the FRET efficiencies of the two conformers are very similar, both conformations could still coexist. In contrast to the results obtained in the presence of Na^+^, application of pressure has only a minor effect on the stability of 22AG and its complex formed with the ThT in the presence of K^+^ (Fig. 3C). Further, it is remarkable that in the presence of both Na^+^ and K^+^, the conformation adopted by the 22AG/ThT complex seems to be largely the same (antiparallel), the pressure dependence being different, however. The antiparallel structure in the ThT complexed state is more pressure stable in the presence of K^+^. This highlights the key role of the internal metal cation in determining the packing properties of the structure and its stability. The stability of the different topologies of GQs at high pressures is also in agreement with results from an earlier study by Sugimoto et al.^57^.

The conformational behavior of 22AG and its complex with ThT was also examined in the presence of the crowding agent Ficoll (at 25 wt%). Here, *E*_rel_ of 22AG in the absence of ThT at 1 bar is ~ 0.42, which is significantly higher than the value obtained in pure buffer. In accordance with the CD data, we can state that also in the presence of Ficoll, the 22AG is mainly unfolded, but a considerable fraction of the quadruplex is folded. In the presence of ThT, *E*_rel_ ≈ 0.64 at ambient pressure, indicating a strong conformational change upon binding, which most likely leads to the population of several conformers: a parallel one and hybrid/antiparallel one. Upon pressurization up to 2000 bar, a significant decrease of *E*_rel_ (from ~ 0.42 to ~ 0.29) is observed in the absence of ThT, i.e. HHP favors the open, unfolded conformation. For the ThT/22AG complex, a substantial pressure dependence of *E*_rel_ was also observed, with *E*_rel_ values decreasing from ~ 0.64 at 1 bar to ~ 0.41 at 2000 bar, indicating a pressure-induced conformational change. According to the *E*_rel_ values reported in Table 2, the parallel conformation would be largely populated in the high-pressure state, similar to the scenario observed for the ThT/22AG complex in pure buffer solution at high pressure. Minor contributions from other conformers (e.g., the unfolded state) cannot be excluded, however.

In principle, the decrease of *E*_rel_ with pressure detected for several of the 22AG/ThT systems could be ascribed to a pressure-induced release of ligand ThT from the 22AG. In other words, pressure could disfavor the formation of the complex. However, this does not seem the case, as it will be shown in the next sections.

### Effect of pressure on the stoichiometry of 22AG/ThT complex

The pressure dependence of the binding constants (*K*_b_) for the complex formation and the four different solution conditions was evaluated by titrating a solution of ThT with a corresponding solution of 22AG and measuring the fluorescence intensity of the fluorophore. Since structural changes of the quadruplex were detected in the FRET experiments, it is mandatory to verify the stoichiometry of the complex also under pressure. This is particularly true for the system in pure buffer and in the presence of Na^+^ and Ficoll. In the K^+^ containing solution it is reasonable to assume that the stoichiometry is still 1:1, since no conformational changes of both 22AG and 22AG/ThT complex were observed. Figures S1, S2 and S3 show the Job’s plot for the complex formation in pure buffer, in the presence of 60 mM Na^+^ and 25 wt% Ficoll at 1, 500, 1000, 1500 and 2000 bar. In pure buffer at 1 bar, on average, 3 ThT molecules bound to 1 22AG molecule were found. At 500 bar, the data suggest that the stoichiometry of the complex is preserved. Beyond 1000 bar, however, a significant change in the intensity at *x*_ThT_ ≈ 0.8 was observed, indicating a change in the stoichiometry of the complex formed. This change is even more evident upon increasing the pressure up to 2000 bar and suggests a change in the stoichiometry from 1:3 to 1:4 (22AG:ThT). The Job’s plot recorded at 1 bar and 500 bar in the presence of 60 mM Na^+^ clearly suggests the formation of a 1:1 complex. Only at pressures at and higher than 1000 bar, a change in the shape of the Job’s plot is observed. An inflection point around 0.7 points to the formation of a 1:2 (22AG:ThT) complex (in an ideal case, the inflection point for a 1:2 complex is located at *x*_ThT_ = 0.67). In the presence of 25 wt% Ficoll, the Job’s plot at 1 bar suggests the formation of a 1:2 (22AG:ThT) complex. The application of pressure up to 500 bar does not cause any change in the stoichiometry. Beyond 1000 bar, the Job’s plots suggest that the stoichiometry of the complex is changing, pointing to the formation of a 1:3 (22AG:ThT) complex at high pressures. This is quite evident at 2000 bar, where an inflection point in the region between *x*_ThT_ = 0.7 and *x*_ThT_ = 0.8 was observed. All the stoichiometries determined are summarized in Table 3.

**Table 3:** Binding constants (*K*_b_) and stoichiometries for the 22AG:ThT complex formation in the indicated solutions and at pressures of 1, 500, 1000, 1500 and 2000 bar (*T* = 25 °C.

Tris 30 mM, pH 7.4			NaCl 60 mM		
*p*/bar	*K*_b_/M^−1^ ∙10^5^	*n*^a^	*p/*bar	*K*_b_/M^−1^ ∙10^5^	*n*^a^
1	0.87 ± 0.06	1:3	1	0.42 ± 0.04	1:1
500	0.97 ± 0.07	1:3	500	0.62 ± 0.02	1:1
1000	1.16 ± 0.07	1:4	1000	0.83 ± 0.02	1:2
1500	1.61 ± 0.19	1:4	1500	1.23 ± 0.03	1:2
2000	2.00 ± 0.02	1:4	2000	1.84 ± 0.01	1:2

KCl 60 mM			Ficoll 25 wt%		
*p/*bar	*K*_b_/M^−1^ ∙10^5^	*n*^a^	*p/*bar	*K*_b_/M^−1^ ∙10^5^	*n*^a^
1	3.51 ± 0.42	1:1	1	1.39 ± 0.07	1:2
500	4.31 ± 0.54	1:1	500	1.19 ± 0.07	1:2
1000	4.29 ± 0.54	1:1	1000	1.18 ± 0.08	1:3
1500	4.82 ± 0.55	1:1	1500	1.35 ± 0.08	1:3
2000	5.00 ± 0.66	1:1	2000	1.58 ± 0.11	1:3

^a^*n* represents the stoichiometry of binding as 22AG:ThT.

### The pressure dependence of the binding constants

**Figure 4:**
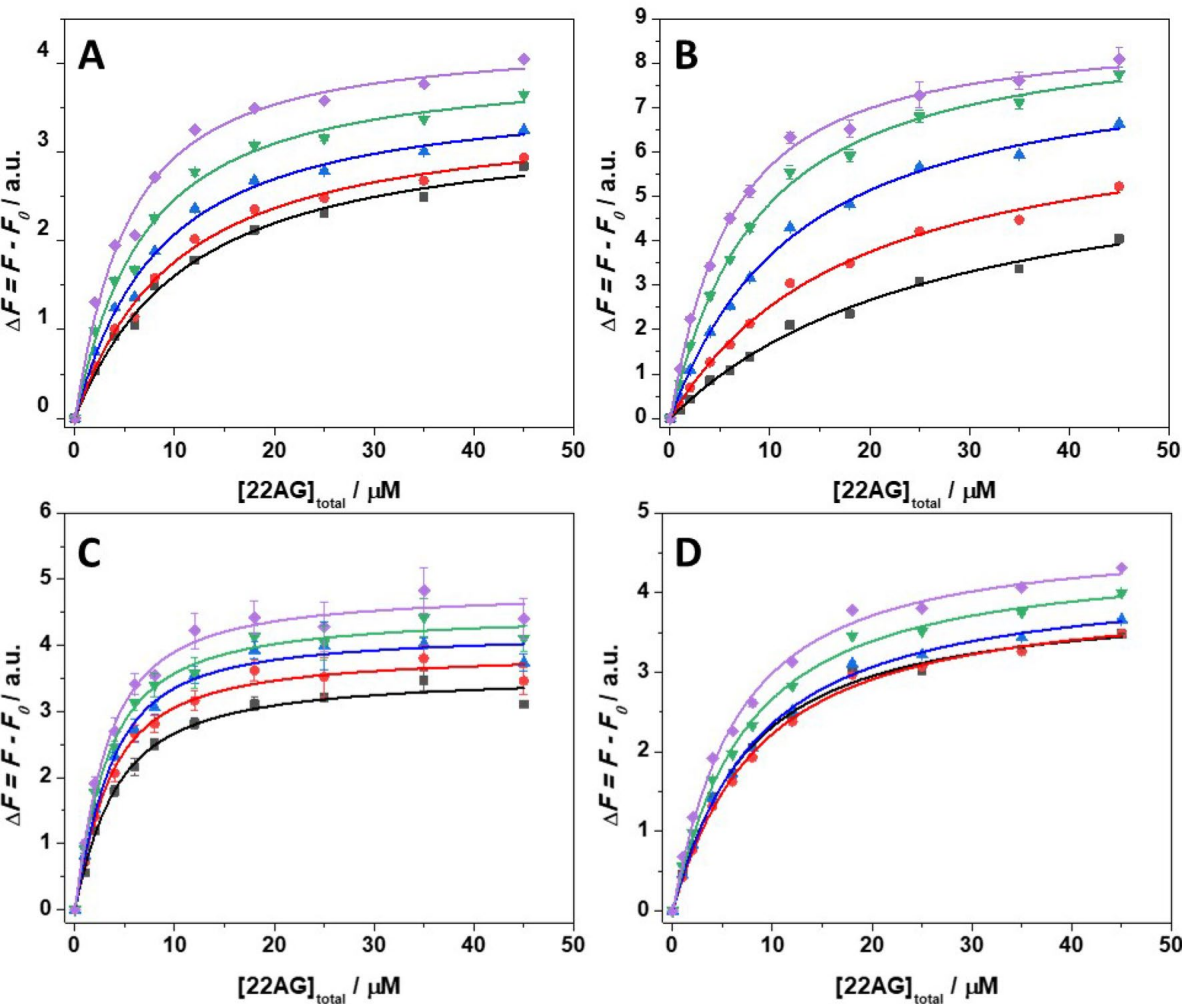
Binding isotherms for the 22AG/ThT system in (**A**) 30 mM Tris, pH 7.4, (**B**) in the presence of 60 mM NaCl, (**C**) in the presence of 60 mM KCl, and (**D**) in the presence of 25 wt% Ficoll at the pressures of: 1 bar (black squares), 500 bar (red circles), 1000 bar (blue triangles), 1500 bar (green reversed triangles), and 2000 bar (violet diamonds). All the experiments were performed in triplicate at the temperature of 25 °C. Where not shown, the error bars are smaller than the symbol size.

With the knowledge of the stoichiometries for the 22AG/ThT complex formation in the whole pressure range from 1 to 2000 bar, it is now possible to determine the binding constants and their pressure dependence, which reflect the strength of interaction between the ligand and the quadruplex. Figure 4 displays the binding curves for the complex formation at all solution conditions as a function of pressure. The binding constants obtained from the data fitting procedure (assuming equivalence among different sites) are reported in Table 3.

**Figure 5:**
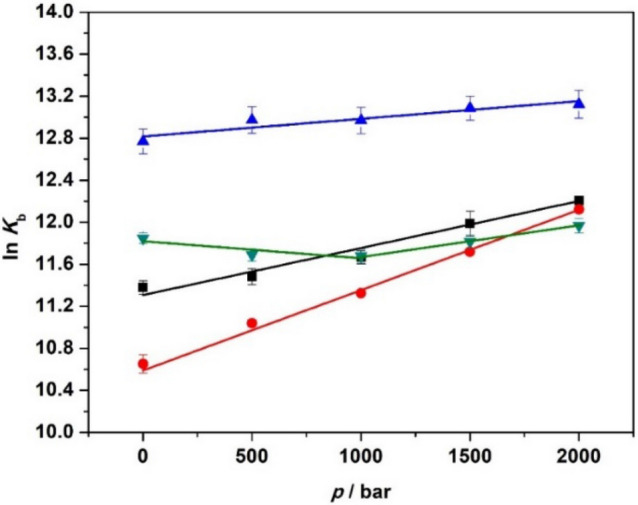
Pressure dependence at *T* = 25 °C of the natural logarithm of the binding constant, ln*K*_b_, in 30 mM Tris pH 7.4 (black squares), in the presence of 60 mM NaCl (red circles), in the presence of 60 mM KCl (blue triangles), and in the presence of 25 wt% Ficoll (green reversed triangles). From the fit of ln*K*_b_ *vs. p,* the reaction volume (Δ*V*_r_) can be determined. Where not shown, the error bars are smaller than the symbol size.

An inspection of Table 3 reveals that in pure buffer and in the presence of Na^+^ the *K*_b_ increases significantly with pressure. In other words, the application of pressure favors the formation of the complex between 22AG and ThT. In the presence of K^+^, only a slight increase of *K*_b_ is detected, revealing that the complex is only marginally affected by pressure. In the Ficoll containing buffer, a biphasic behavior is observed, instead. A small decrease of *K*_b_ in the 1–1000 bar pressure range is followed by a more pronounced increase up to 2000 bar. From the pressure dependence of ln(*K*_b_), it is possible to estimate the volume change of the ligand binding reaction, Δ*V*_r_, which represents the partial molar volume difference between the complexed (ThT bound to 22AG) and the uncomplexed (ThT + 22AG) states, i.e., Δ*V*_r_ = *V*_complex_—*V*_ThT+22AG_^46,58^. If the complex occupies a smaller volume with respect to the uncomplexed states, the binding constant increases with pressure and the reaction volume will be negative, and vice versa. In analysing our binding data, we employed an equivalent and independent sites binding model. In this model, it is assumed that all the ThT molecules bind to 22AG with the same affinity (represented by the binding constant, *K*_b_). Thus, the volume changes (∆*V*_r_) are reported per mol of 22AG. In case of mixed populations, an apparent ∆*V*_r_ is measured only, which represents an average over the different populations with their different ∆*V*_r_'s*.* Figure 5 shows all ln(*K*_b_) *vs. p* plots and Table 4 summarizes the Δ*V*_r_ values for all solution conditions.

**Table 4:** Reaction volumes (Δ*V*_r_) for the complex formation between 22AG and ThT at the temperature of 25 °C. The Δ*V*_r_ values were obtained from the slope of ln*K*_b_ *vs. p*, which yields—Δ*V*_r_/(*RT*).

Solvent	Δ*V*_r_/mL mol^−1^
30 mM Tris, pH 7.4	− 11.2 ± 0.7
+ 60 mM NaCl	− 19.1 ± 0.6
+ 60 mM KCl	− 4.2 ± 0.8
+ 25 wt% Ficoll	4.0 ± 2.1/− 7.2 ± 0.3^a^

^a^The first value and the second value refer to the volume change in the 1–1000 bar and 1000–2000 bar pressure range, respectively.

In pure buffer, a negative value of Δ*V*_r_ was determined, indicating that the application of pressure will favor the formation of the complex, i.e. the volume occupied by the complex state is lower with respect to the volumes occupied by the nucleic acid and the ligand in the uncomplexed state. In the presence of Na^+^, a negative reaction volume was obtained as well, but the value is about twice as high as in pure buffer. Instead, in the presence of K^+^ cations, the volume change is quite small, highlighting that the formation of the complex is only marginally affected by pressure. Interestingly, in the presence of the crowder Ficoll, the ln*K*_b_ *vs. p* plot exhibits a biphasic behavior: a decrease in the range 1–1000 bar and an increase in the range between 1000 and 2000 bar. Obviously, two opposite trends partially compensate each other, rendering the overall pressure dependence of the species involved quite small.

## Discussion

Using FRET and CD spectroscopy, Job's plot and binding equilibrium measurements, we investigated the effect of pressure on the binding reaction of the ligand ThT to the quadruplex 22AG in solutions containing different ionic species (NaCl, KCl) and a crowding agent (Ficoll).

Our data show that the binding strength, quantitatively described by *K*_b_, and the stoichiometry of complex formation as well as the conformational substates of the quadruplex depend strongly on the solution conditions. At ambient pressure and 25 °C, the CD and FRET data indicate that the 22AG is predominantly in an unfolded conformation in neat buffer and in the presence of the crowding agent Ficoll. Instead, in the presence of Na^+^ and K^+^, the 22AG takes on mainly the folded antiparallel and hybrid-1 conformation, respectively. Upon addition of ThT, the 22AG strand adopts mainly a parallel conformation in neat buffer and in the presence of Ficoll, with a small population of hybrid/antiparallel structures in the latter case. Instead, an antiparallel conformation is taken in the presence of the monovalent cations Na^+^ and K^+^. Thus, the structure adopted upon ThT binding depends on the initial 22AG conformation. It is important to recall that the ThT molecule, at pH of 7.4, is positively charged. Hence, next to the interaction with Na^+^ and K^+^ cations, electrostatic interactions of ThT with the negatively charged phosphate backbone of the oligonucleotide besides π-stacking with the guanine planes can be expected, which leads to the various conformational changes observed and the likelihood to occupy different binding sites.

Surprisingly, the stoichiometry of binding also depends on the initial conformation of the GQ. The stoichiometry is 1:1 (22AG:ThT) in the presence of Na^+^ and K^+^ cations, i.e. when the 22AG is folded in a compact structure at ambient pressure. Instead, in pure buffer and in the presence of Ficoll, the stoichiometries estimated are 1:3 and 1:2, respectively. Through ESI–MS experiments, up to 4 ThT molecule bound to one 22AG were observed in 100 mM NH_4_OAc, indicating that multiple binding modes are in fact possible^30^. Even if the exact location of these sites is not exactly known, we can speculate that the interaction occurs through stacking of ThT molecules with the guanine planes. In the parallel conformation present in buffer (next to the hydrid/antiparallel one), both guanine planes have been shown to be accessible to the ThT^52^, opening up the possibility of several binding modes. Such scenario would be in agreement with the Job’s plot data (Fig. 1) for 22AG in 30 mM Tris, pH 7.4, and in the presence of Ficoll. Since in pure buffer a 1:3 (22AG:ThT) complex was observed, a ThT groove binding or intercalation cannot be excluded a priori*.* In fact, groove binding to the parallel conformation is also supported by docking studies^5^. Instead, in the presence of Na^+^ and K^+^, the ThT is bound to the antiparallel conformation. An inspection of the structure of this conformation^52^ reveals that there could be two possible binding modes on the G-planes. However, one G-plane is surrounded by two thymine-rich loops that, owing to their flexibility, can exert steric hinderance, preventing the binding in that region. The opposite G-plane presents, instead, only one loop (of sequence ATT). This loop should be more rigid than the other two loops so that ThT can easily access this site. This scenario is also supported by simulations where binding at this G-plane was observed^5^.

The pressure dependent data revealed that, depending on the different solution conditions, the application of high pressure can dramatically affect both the binding affinity and stoichiometry. This phenomenon is the result of the combined influence that pressure has on the conformational landscape of the 22AG sequence and on the binding properties themselves. Generally, it was found that the binding constant *K*_b_ increases significantly with pressure, i.e., the application of pressure favors the formation of the ThT-22AG complex. Thus, the complex occupies a smaller partial molar volume with respect to the uncomplexed states. The corresponding volume changes depend on the solution conditions, however. The FRET data reported (Fig. 3) clearly show that, with the exception of the particularly stable K^+^ containing system, the initial structure of the 22AG sequence changes upon pressurization, as previously observed for 22AG and other sequences^15,36^. Thus, the reaction volumes (Δ*V*_r_) reported in Table 4 are volume changes that also include the conformational transition volumes (Δ*V*_tr_) of the nucleic acid strand, i.e., Δ*V*_r_ = Δ*V*_tr_ + Δ*V*_b_, where Δ*V*_b_ is the volume change accompanying the binding of ThT, only. Further, in case of mixed populations, the Δ*V*_r_ data represent an average over the various contributions of the different conformers.

In neat buffer, the 22AG sequence is essentially unfolded at all pressures. In the presence of ThT, 22AG acquires mostly a parallel conformation that is preserved even up to 2000 bar. The volume change for the transition from an unfolded to parallel conformation was determined for the c-MYC sequence^59^ and it was found to be Δ*V*_tr_ = 16 mL mol^−1^. Since Δ*V*_r_ = − 11 mL mol^−1^, the volume change for the binding of ThT only may be determined to Δ*V*_b_ ≈ − 27 ± 1.9 mL mol^−1^. Of note, even if the structure of 22AG/ThT is largely preserved, minor changes in *E*_rel_ were observed upon pressurization in the FRET data, indicating that the 22AG adopts a slightly opened, but still largely folded conformation. This observation may give an indication for the change in the binding stoichiometry from about 3 to 4 as inferred from the Job’s plot data.

In the Na^+^ containing buffer, the FRET data clearly show that the 22AG sequence in the antiparallel conformation is not pressure stable. In fact, the transition volume from the antiparallel conformation to the unfolded (coil) state was found to be around -56 mL mol^−1^ in 50 mM NaCl^37,38^, i.e. pressure favors the more open, unfolded state with respect to the antiparallel one. Upon binding to ThT, at 1 bar, no significant conformational changes were observed. The situation changes at high pressure. At 2000 bar, a value of *E*_rel_ ≈ 0.40 is recorded, suggesting more parallel (and/or unfolded) conformers for the 22AG (Table 2). Hence, the binding of ThT to 22AG at high pressure seems to promote the transition to the parallel conformation. As reported above, Δ*V*_tr_ for the coil-to-parallel transition amounts to 16 mL mol^−1^. With Δ*V*_r_ = − 19.1 mL mol^−1^ (Table 4), we obtain for the binding volume Δ*V*_b_ ≈ − 35 ± 1.8 mL mol^−1^. Thus, the binding of ThT has to compensate the unfavorable (positive) Δ*V*_tr_ for the coil-to-parallel transition. The Job’s plot suggests that at and beyond 1000 bar, on average 2 ThT molecules are bound to one 22AG. In fact, according to the reported structure of the parallel conformation, two binding modes can be expected since the two G-planes should be easily accessible to the ThT molecule, which might help in stabilizing the structure.

In the presence of K^+^ cations, the 22AG sequence is folded in the hybrid-1 conformation. In the pressure range covered, the hybrid-1 conformation is rather stable, as inferred from the minor changes detected in the pressure-dependent FRET data (Fig. 3C). However, at higher pressures, the unfolded state prevails, and the Δ*V*_tr_ value for the hybrid-1-to-coil transition was found to be about − 19 mL mol^−1^^60^. For the longer sequence Tel26, a negative transition volume was also found^61^. Upon ThT binding, the 22AG sequence adopts an antiparallel conformation that remains essentially unperturbed up to 2000 bar. The volume change related to the hybrid-1-to-antiparallel conformational transition can be estimated from the Δ*V*_tr_ for the coil-to-antiparallel and the coil-to-hybrid-1 transitions, which amount to 56 mL mol^−1^ and 19 mL mol^−1^, respectively^38,60^, leading to Δ*V*_tr_ ≈ 37 mL mol^−1^ for the hybrid-1-to-antiparallel transition. This indicates that the antiparallel conformation is less compact with respect to the hybrid-1 conformation and should hence be less pressure stable. According to Table 4, Δ*V*_r_ is − 4.2 mL mol^−1^, meaning that the volume change related to the binding of ThT only, Δ*V*_b_, should be − 41 ± 4.7 mL mol^−1^. Again, the strong and negative Δ*V*_b_ has to counterbalance the unfavorable conformational transition volume, Δ*V*_tr_.

These negative binding volumes estimated for the ligand binding reaction in pure buffer (i.e., in the absence of cations), Na^+^ and K^+^ solutions may be expected to result from the release of packing defects (void volume) at the binding site upon external docking of the ThT molecules to the G-tetrad, which must compensate for a volume increase upon dehydration of the guanines in the G-tetrad plane and the ThT molecules upon binding, if there is any. It is interesting to note that for the binding of hemin to 22AG, a slightly positive and a negative binding volume was found in the presence of Na^+^ and K^+^ cations, respectively^57^, demonstrating that the environmental conditions can affect the hydration level of ligands as well. The binding volumes of the different systems are of similar magnitude. Small differences in Δ*V*_b_ values will be due to differences of void volume at the docking sites. Please note that the volume changes are rather small, about the size of about 2 water molecules only (the partial molar volume of water is 18 mL mol^−1^). Hence, binding will most likely occur with little water released at the binding site, because the release of a significant amount of bound water would lead to positive Δ*V*_b_ values as the density of hydration water is generally larger compared to bulk water^6,37^.

The presence of crowding agents can have a marked impact on the structure and pressure-stability of GQs. Generally, changes in the topology of the GQ's conformation in the presence of crowders may originate from conformation-sensitive binding of the crowding agent to the GQ and/or the combined effects of excluded volume and differential hydration of the GQ. Previously, it was shown that the human telomeric G-quadruplex in 15 wt% Ficoll is pressure stable up to 1000 bar, but that at higher pressures, the parallel conformation is favored over the antiparallel one^15^. Of note, self-crowding of the DNA was also shown to influence the stability of quadruplexes. For the 22AG, the Δ*V*_tr_ towards to unfolded state is negative in dilute solution, but becomes positive for self-crowding conditions, indicating that unfolding is not promoted by pressure anymore^60^. In the present case, in 25 wt% Ficoll, our FRET data show that the 22AG sequence is mainly unfolded at ambient pressure, with a certain amount of hybrid/antiparallel conformers being also present as deduced from the CD experiments. At high pressure (2000 bar), the GQ is most likely completely unfolded. The addition of ThT causes a conformational change of the DNA strand that adopts mainly a parallel conformation now. However, the CD data clearly suggest that other conformations, like hybrid or antiparallel ones, are present in solution as well. Hence, several conformations are populated at these strong crowding conditions, which opens up the possibility of filling multiple binding sites. Thus, the decrease (1–1000 bar) and the subsequent increase (1000–2000 bar) of the *K*_b_ value can be explained by different 22AG conformers present in the crowded solution that exhibit different binding volumes and pressure stabilities, with Δ*V*_tr_ values being positive for the unfolded-to-parallel transition (16 mL mol^−1^) and slightly negative for the hybrid-1-to-parallel transition (~ − 3 mL mol^−1^), according to literature data^59,60^.

## Summary and concluding remarks

Much effort has been invested in recent years in the quest for drugs that would lock G-rich regions of DNA into G-quadruplex conformations, thereby, for example, inhibiting oncogene expression and telomere synthesis. Small organic molecules designed to bind to GQs are, generally, like ThT, positively charged aromatic molecules and may interact with the GQ via external stacking and/or intercalation. A comprehensive understanding of the binding of small molecule ligands to GQs is therefore of paramount importance. Pressure modulation was used here to help identify the different conformational substates adopted by the 22AG quadruplex at the different solution conditions in the absence and presence of the ligand, and to determine the volumetric changes upon complex formation and of the conformational transitions involved. Altogether, these pressure-axis experiments helped us shed more light on the forces and mechanisms involved in the ligand binding process at the different solution conditions and at high pressure.

**Figure 6:**
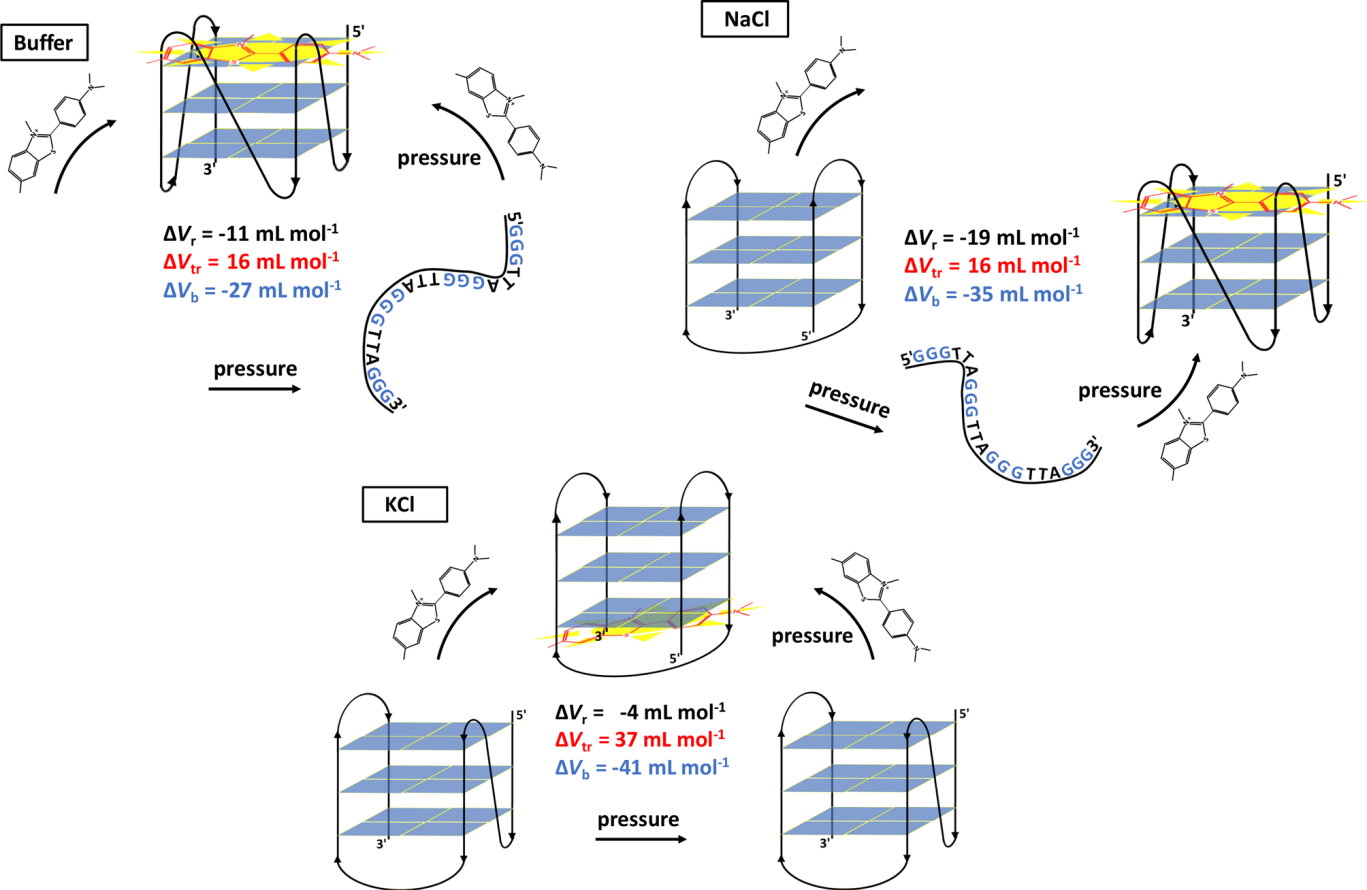
Schematic representation of the effect of different solution conditions (pure buffer and different salts (60 mM NaCl, KCl)) and pressure on the conformation of the 22AG quadruplex in the absence and presence of the ligand ThT. Owing to the complexity of the system, no sketch of the Ficoll data are shown here. For sake of clarity, the possibility of multiple binding modes (as mainly occurring at high pressures) is not taken into account in this schematic, only one representative binding site is shown here. The (apparent) volume changes for the overall reaction (Δ*V*_r_) and tentative values of the ThT binding step (Δ*V*_b_) and the conformational transitions (Δ*V*_tr_) of the GQ are also indicated.

Figure 6 summarizes our findings in a schematic representation. Our results show that the conformational substates of the GQ, as well as the binding strength and the stoichiometry of ThT/22AG complex formation, depend strongly on the solution conditions. Further, G-quadruplexes may even form without the stabilizing influence of a centrally bound cation upon drug binding. Hence, these data provide compelling evidence for a distinct and rugged energy and conformational landscape of the system. Upon addition of ThT, the 22AG strand mainly adopts a parallel conformation in pure buffer and in the presence of the crowding agent Ficoll, with a small population of hybrid/antiparallel structures in the latter case. Instead, an antiparallel conformation is adopted in the presence of the monovalent cations Na^+^ and K^+^. It is noteworthy that the stoichiometry of binding also depends on the initial conformation of the GQ. Generally, it was found that the binding constant *K*_b_ increases significantly with pressure, i.e., the application of pressure favors the formation of the ThT/22AG complex. Hence, the complex occupies a smaller partial molar volume compared to the uncomplexed state. Depending on the different solution conditions, HHP can affect both the binding affinity and stoichiometry. This phenomenon is the result of the combined influence that pressure exerts on the conformational landscape of the 22AG sequence and on the binding properties themselves. Thus, the (apparent) reaction volumes (Δ*V*_r_) determined from the pressure dependent apparent binding constants are volume changes that include both the conformational transition volumes of the nucleic acid strands and the corresponding volume changes associated with the binding events of ThT. The magnitude of the binding volumes is a hallmark of the packing defects and hydrational changes upon ligand binding.

Overall, these pressure-axis experiments helped us to shed more light on the forces and mechanisms involved in the ligand binding process of the GQ at different solution conditions and at high pressure. Such a pressure perturbation approach could be used in further studies to quantify and optimize G-quadruplex-ligand interactions. Finally, there is also a biologically relevant aspect to these studies. HHP can impact DNA structures such as G-quadruplexes inside living cells and thus affect expression of genetic information in deep sea organisms. Here we show that hydrostatic pressure at multi-hundred bar levels can affect not only the conformational dynamics and structure of GQs, but also their ligand binding reactions. As in the case presented here, an increase of pressure can actually be beneficial and lead to an increase in the binding constant.

## Supplementary Information

Supplementary Information.
