# Supplementary material for: Unraveling the binding characteristics of small ligands to telomeric DNA by pressure modulation
Source: Sci Rep. 2021 May 6;11:9714. doi: 10.1038/s41598-021-89215-2 (PMC8102477; doi:10.1038/s41598-021-89215-2)
Supplement: Supplementary file 1 — Supplementary Information. [file 41598_2021_89215_MOESM1_ESM.docx]

**Supplementary Information**

**Unraveling the binding characteristics of small ligands to telomeric DNA by pressure modulation**

Rosario Oliva*, Sanjib Mukherjee, and Roland Winter*

Physical Chemistry I - Biophysical Chemistry, Faculty of Chemistry and Chemical Biology, TU Dortmund University, Otto-Hahn Strasse 4a, D-44227 Dortmund, Germany.

**Additional Figures**


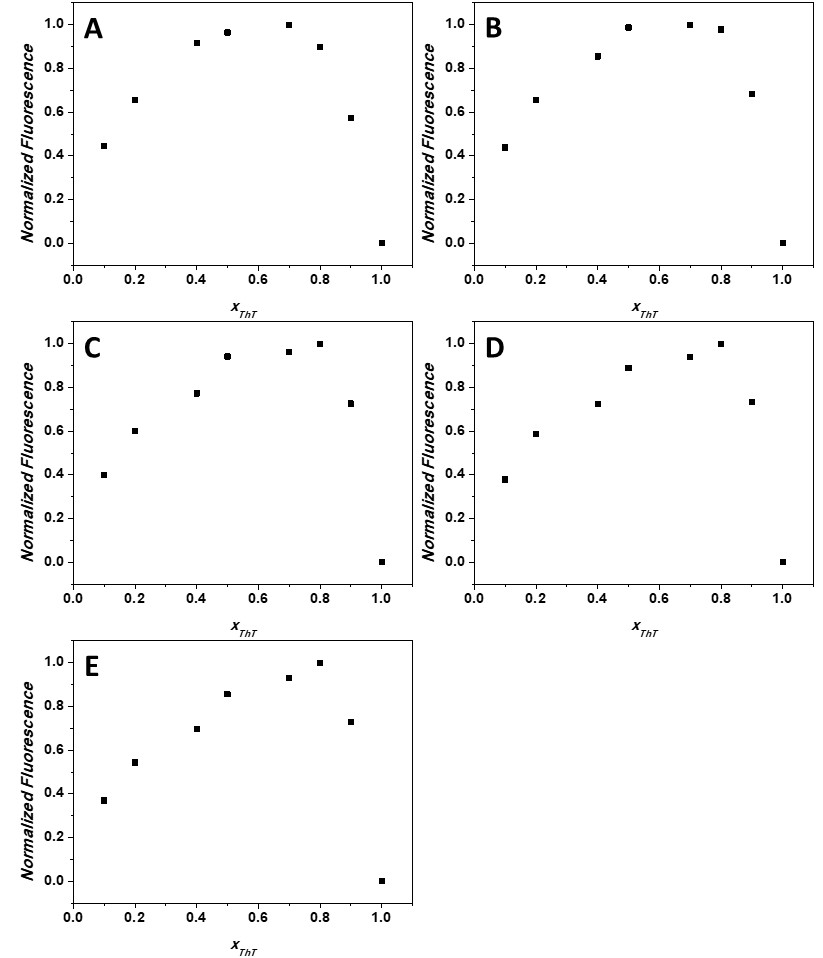


**Figure S1.** Job’s plots for the 22AG/ThT system in 30 mM Tris, pH 7.4, obtained by means of high pressure fluorescence spectroscopy at *T* = 25 °C and at the pressures of (A) 1 bar, (B) 500 bar, (C) 1000 bar, (D) 1500 bar, and (E) 2000 bar. The total concentration ([ThT]+[22AG]) was 35 µM. Where not shown, the error bars are smaller than the symbol size.


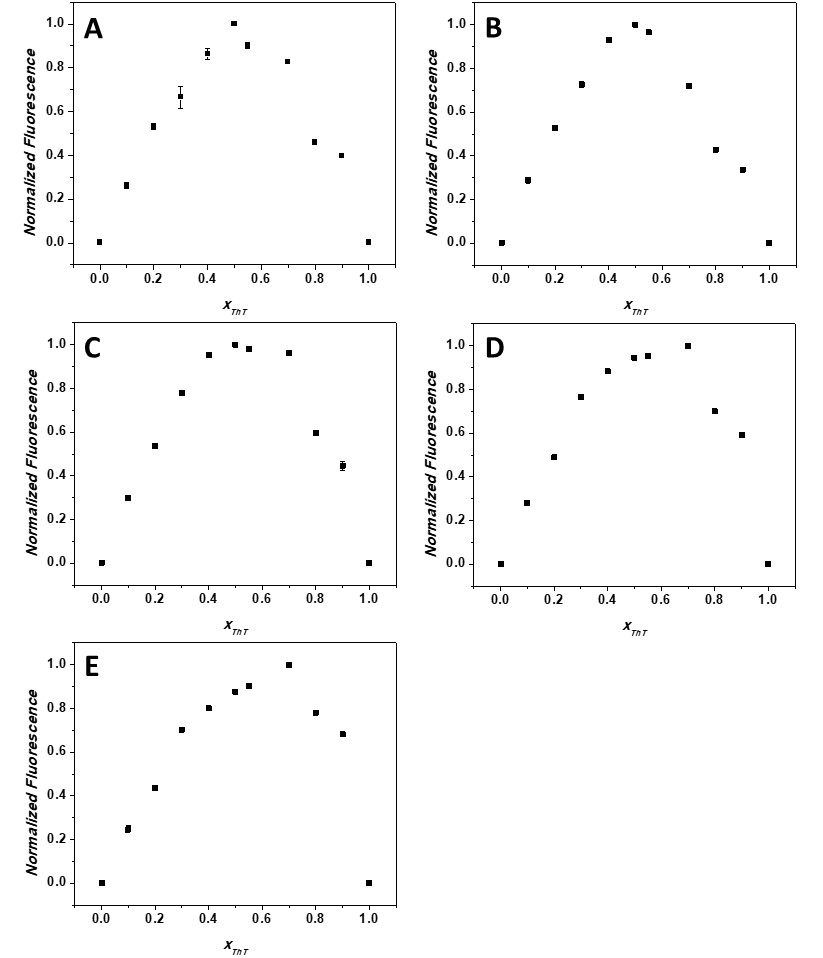


**Figure S2.** Job’s plots for the 22AG/ThT system in 30 mM Tris, 60 mM NaCl, pH 7.4 obtained by means of high pressure fluorescence spectroscopy at *T* = 25 °C and at the pressures of (A) 1 bar, (B) 500 bar, (C) 1000 bar, (D) 1500 bar and (E) 2000 bar. The total concentration ([ThT]+[22AG]) was 35 µM. Where not shown, the error bars are smaller than the symbol size.


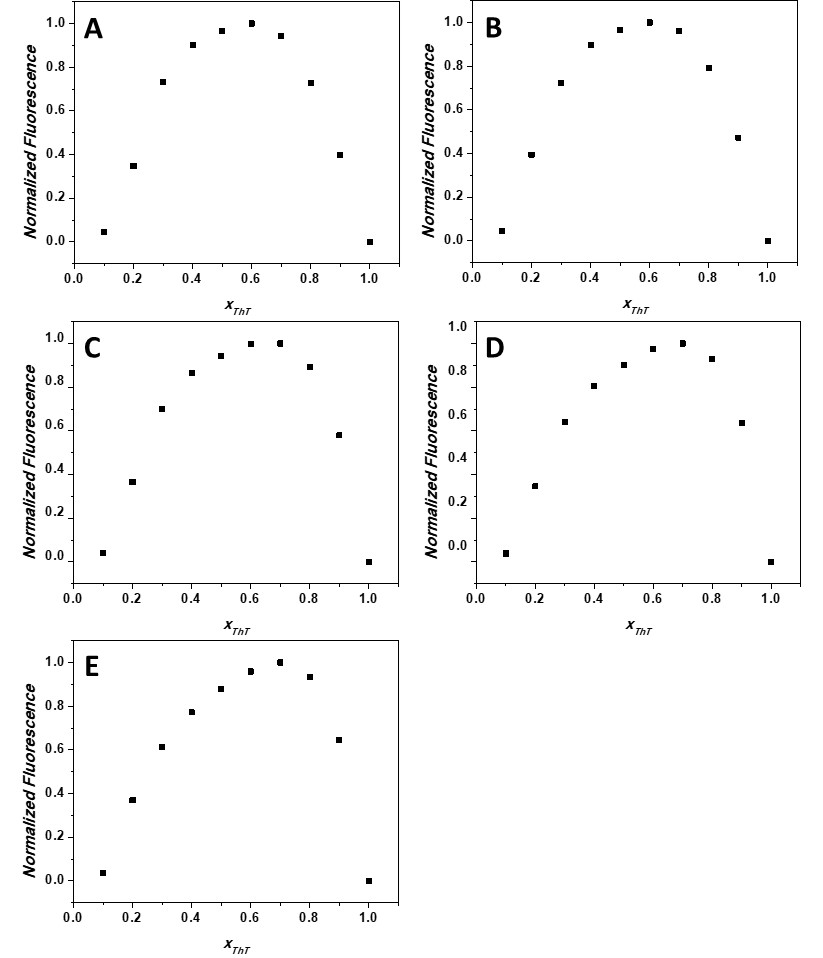


**Figure S3.** Job’s plots for the 22AG/ThT system in 30 mM Tris, 25 wt% Ficoll, pH 7.4 obtained by means of high pressure fluorescence spectroscopy at *T* = 25 °C and at the pressures of (A) 1 bar, (B) 500 bar, (C) 1000 bar, (D) 1500 bar, and (E) 2000 bar. The total concentration ([ThT]+[22AG]) was 35 µM. Where not shown, the error bars are smaller than the symbol size.
